# Association between reproductive history and chronic low back pain in the US women: a cross-sectional study

**DOI:** 10.7189/jogh.16.04238

**Published:** 2026-08-28

**Authors:** Yihui Zhang, Lixin Zhao, Dexin Hu, Xueru Shen, Xiaoxue Dong, Yuheng Lu

**Affiliations:** 1 Department of Rehabilitation Therapeutics, School of Population Health, Tianjin College, University of Science and Technology Beijing, Tianjin, China; 2 Department of Rehabilitation Medicine, Xijing Hospital, Fourth Military Medical University, Xi’an, China; 3 Department of Sports Rehabilitation, School of Sports and Health Science, Xi’an Physical Education University, Xi’an, China; * Joint first authorship.

**Keywords:** chronic low back pain, reproductive history, gravidity, parity, age at first live birth

## Abstract

**Background:**

Low back pain (LBP) is more prevalent in women, particularly peripartum, suggesting a link between reproductive history and chronic LBP (CLBP). Nationally representative evidence on this association is limited.

**Methods:**

We conducted a cross-sectional study using NHANES 1999–2004 and 2009–2010 data. Weighted multivariable logistic regression examined associations between reproductive factors and CLBP. Restricted cubic splines assessed dose-response relationships. Subgroup and sensitivity analyses ensured robustness; multiple imputations addressed missing data.

**Results:**

Women with CLBP had a significantly younger age at first live birth (22.01 *vs.* 23.05 years, *P* < 0.001) and elevated gravidity and parity. In fully adjusted models, both gravidity (odds ratio (OR) = 1.08; 95% confidence interval (CI) = 1.05–1.10) and parity (OR = 1.07; 95% CI = 1.04–1.11) were positively associated with CLBP, while older age at first (OR = 0.96; 95% CI = 0.94–0.97) and last live birth (OR = 0.97; 95% CI = 0.95–0.98) were associated with lower odds of CLBP. The highest odds were observed in women with a history of pregnancy but no live birth (OR = 2.33; 95% CI = 1.71–3.16). Birth at age ≤16 years was associated with 66% higher odds of CLBP (OR = 1.66; 95% CI = 1.26–2.18).

**Conclusions:**

First live birth, higher gravidity and parity, earlier last parity, and pregnancy without live birth are independently associated with a higher CLBP prevalence in women. Integrating reproductive history into pain-related clinical assessment and preventive care may be beneficial for targeted CLBP screening and prevention.

Low back pain is one of the leading causes of disability worldwide [1,2] and its high prevalence and recurrence rate have caused a severe socioeconomic burden. Among all populations, the prevalence of low back pain is generally higher in women than in men [3,4] and it is particularly common during pregnancy and the postpartum period [5]. Studies have shown that 50%–75% of pregnant women experience varying degrees of back pain during pregnancy [6], with symptoms persisting in some individuals postpartum and even progressing to chronic pain, significantly affecting their quality of life and work force participation. However, there is currently a lack of systematic and representative epidemiological studies investigating whether women's unique reproductive experiences contribute to long-term effects on chronic low back pain (CLBP).

From a physiological perspective, pregnancy and childbirth are associated with hormonal fluctuations (such as relaxin and oestrogen) [6,7] and biomechanical changes (including ligament relaxation and increased spinal load) [7,8] which can have a profound may significantly influence on spinal stability and intervertebral disc structure. Although this mechanism has long been a focus of attention, and studies have suggested that full-term pregnancy and parity may be associated with an increased risk of CLBP, the conclusions in the literature are not consistent [9–11]. Some studies have observed that women with high-frequency childbirth have a lower prevalence of CLBP, suggesting that this association may exhibit a nonlinear distribution. More importantly, existing research has largely focused on pregnancy itself, while pregnancy without live birth (including miscarriage, induced abortion, ectopic pregnancy, and stillbirth) has received relatively little attention. It should be noted that this category represents is a heterogeneous condition, which may occur at different gestational stages and vary substantially in clinical presentation. Some non-live birth outcomes may involve minimal physiological changes, whereas others may be accompanied by experiences more similar to labor or delivery, and may include both spontaneous and induced events. Given this variability, the potential pathways linking these outcomes to chronic low back pain may differ across individuals. However, due to data limitations in large population-based surveys such as NHANES, these diverse events are often grouped into a single category, and their associations with CLBP remain insufficiently studied.

In the US, reproductive experiences among women are highly heterogeneous, encompassing wide variation in age at childbirth, number of pregnancies and live births, and pregnancy outcomes. While pregnancy-related low back pain has been well documented in peripartum populations, existing studies in the US have largely focused on single reproductive events or short-term outcomes, with limited attention to women’s cumulative reproductive history across the life course. Moreover, prior research on chronic low back pain has primarily examined demographic, occupational, and lifestyle factors, whereas reproductive factors beyond pregnancy itself remain underexplored. Given the substantial burden of CLBP among US women and the lack of effective primary prevention strategies, examining reproductive history within a nationally representative US population may provide important insights into sex-specific pathways of CLBP vulnerability.

Therefore, using data from the National Health and Nutrition Examination Survey (NHANES) 1999–2004 and 2009–2010, this study examined the associations between key reproductive factors – including gravidity, parity, pregnancy outcomes, and reproductive timing – and chronic low back pain in a nationally representative sample of the US women. We further evaluated potential dose-response and nonlinear relationships to better characterise how cumulative reproductive exposure across the life course may relate to CLBP prevalence.

Based on existing evidence that reproductive events involve sustained hormonal, biomechanical, and inflammatory changes that may affect spinal structures, we hypothesised that cumulative reproductive exposure and earlier reproductive timing would be associated with a higher prevalence of chronic low back pain. In addition, pregnancy without achieving live birth may confer elevated CLBP risk due to combined physiological stress and psychological burden. Accordingly, this study examined the associations between reproductive history and CLBP in a nationally representative sample of the US women.

**Adherence to JoGH’s Guidelines for Reporting Analyses of Big Data Repositories Open to Public (GRABDROP)**

This study adheres to the Journal of Global Health's Guidelines for Reporting Analyses of Big Data Repositories Open to the Public (GRABDROP) [12]. Detailed information regarding adherence to these guidelines is provided in Table S1 in the **Online Supplementary Document**.

## METHODS

### Study analysis

This study employed a cross-sectional design using data from the National Health and Nutrition Examination Survey (NHANES). NHANES is conducted by the National Center for Health Statistics (NCHS) and applies a stratified, multistage probability cluster sampling design to assess the health and nutritional status of the non-institutionalised US population. The NCHS Research Ethics Review Committee approved the NHANES study, and all participants provided written informed consent before participation. Data are publicly available on the NHANES website. This study adhered to the STROBE (Strengthening the Reporting of Observational Studies in Epidemiology) Statement.

Since low back pain data were only collected for individuals aged 20 years and older during the NHANES cycles of 1999–2004 and 2009–2010, this study focused on female participants aged 20 and older from these periods. The cycles from 2005 to 2008 were excluded because the specific survey questions used to define chronic low back pain in this study were not administered during those years, which would have resulted in inconsistent outcome measurement. Participants were excluded from the analysis under the following conditions:

• missing low back pain data;

• missing information about reproductive factors (such as gravidity, parity, age at first live birth, and age at last live birth); and

• missing covariate data.

**Figure 1:**
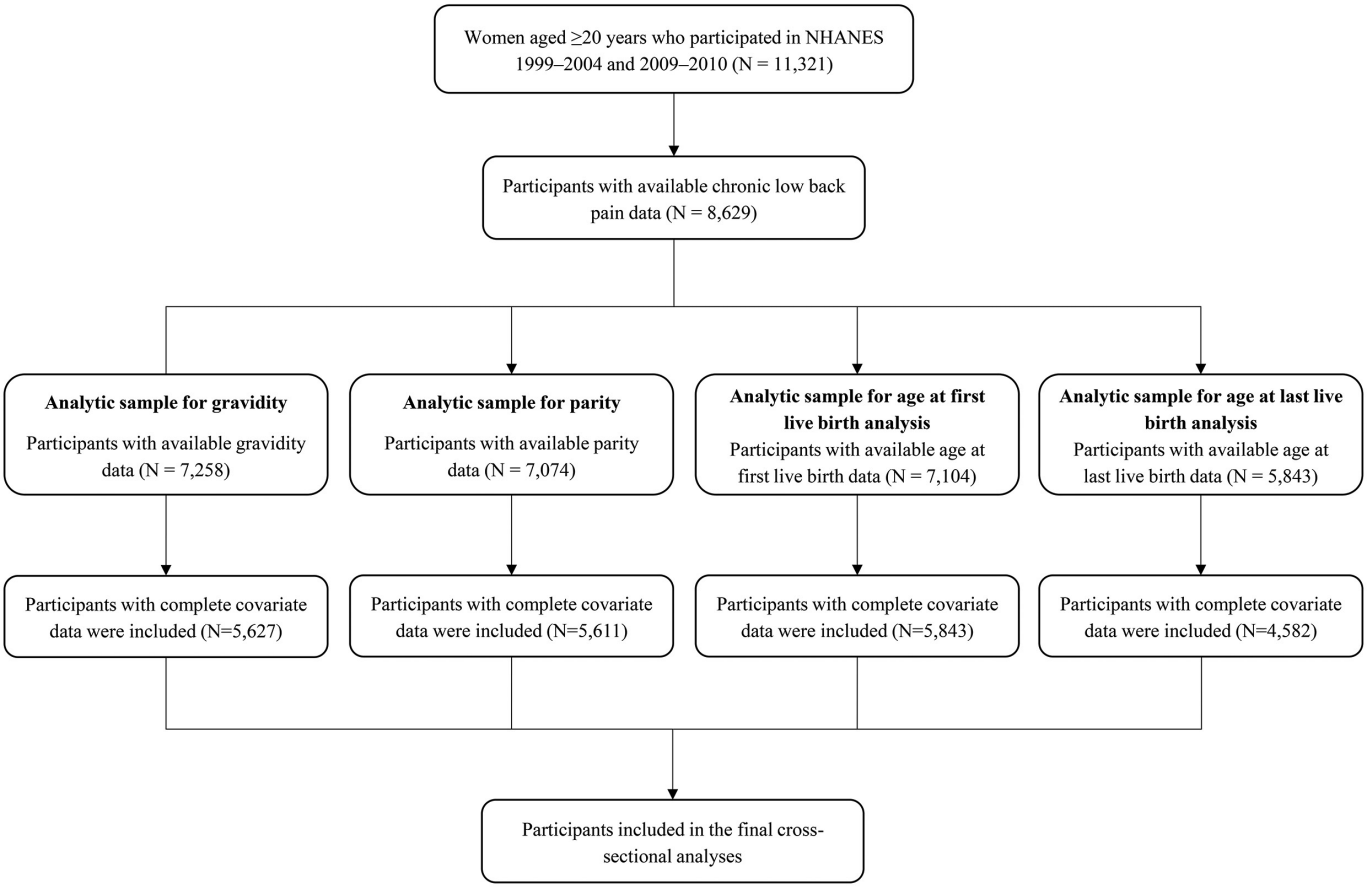
Flowchart of participant selection from the National Health and Nutrition Examination Survey (NHANES) 1999–2004 and 2009–2010.

The process for including and excluding participants is illustrated in **Figure 1**.

### Measures

#### Reproductive factors

This study focused on four reproductive variables: gravidity, parity, age at first live birth, and age at last live birth. These data were collected through a reproductive health questionnaire administered by interviewers trained at mobile examination centres (MECs) using a computer-assisted personal interview system as part of the MEC interview. Participants were asked, ‘Have you ever been pregnant?’ If they answered ‘yes’, they were then asked, ‘How many times have you been pregnant?’ Gravidity was defined as all pregnancy experiences (including current pregnancy, natural delivery, miscarriage, stillbirth, tubal pregnancy, or induced abortion). The question regarding parity was, ‘How many pregnancies resulted in live births?’ This was determined by counting the number of pregnancies that resulted in live births, not the actual number of live-born children. The questions used to determine the age at first live birth and age at last live birth were ‘How many times have you been pregnant? ‘How old were you at the time of your first live birth?’ and ‘How old were you at the time of your last live birth?’ To more accurately explore the association between CLBP and age at live birth, the relevant variables were adjusted as follows: age at first live birth was analysed among women who had at least one live birth. Age at last live birth was restricted to women with two or more live births, as this variable is not defined for women with only one live birth. Women with a single live birth contributed only to the analysis of age at first live birth and were excluded from analyses involving age at last live birth. The four variables were grouped as follows: Gravidity was divided into 0, 1, 2–3, and ≥4 (with the first group as the reference group), and Parity was defined as the number of live births reported by participants [10]. Women with no history of pregnancy were categorised separately as gravidity = 0, while those with at least one pregnancy were further classified according to parity (0, 1, 2–3, and ≥4 live births) [13]. Age at first live birth is divided into five age groups, calculated in years: ≤16, 17–21, 22–26, 27–31, and ≥32, with 22–26 as the reference group [14]; age at last live birth is similar, divided into ≤24, 25–30, 31–35, and ≥35 [15], with 25–30 as the reference group.

#### Outcome

Chronic low back pain was defined based on participants’ self-reported history of low back pain lasting three consecutive months or longer, as assessed in the Arthritis Questionnaire (ARQ). Specifically, participants were asked whether they had experienced back pain every day for three months or longer during the past 12 months. This 12-month period prevalence definition is consistent with the National Institutes of Health (NIH) Chronic Low Back Pain Research Standards Working Group definition, which emphasises pain lasting at least three months [16].

#### Covariates

The covariates were divided into three domains. First, demographic characteristics: age, race, poverty income ratio (PIR) [17], marital status, educational attainment, and body mass index (BMI). Poverty income ratio was calculated by the National Center for Health Statistics as the ratio of family income to the federal poverty threshold, adjusted for family size and survey year, with higher PIR values indicating higher household socioeconomic status. Second, lifestyle: smoking status [18], alcohol consumption [19], and physical activity [20,21]. Third, health conditions: diabetes [18], cardiovascular disease [22], hyperlipidaemia [23], and hypertension [24]. The basis for the diagnosis and classification of all covariates can be found in Table S2 in the **Online Supplementary Document**.

### Analysis plan

All analyses included sampling weights, stratification, and primary sampling units to provide reliable national estimates. Continuous variables are presented as weighted means (standard errors) or medians (interquartile ranges). Categorical variables are presented as weighted counts or percentages. Weighted *t* tests, one-way analysis of variance (ANOVA) tests (for continuous variables), and χ^2^ tests (for categorical variables) were used to assess differences between participants with chronic low back pain and those without, or between different reproductive factor groups. The selection of weights used for analysis was based on the instructions provided in the NHANES database [25]. Following the National Center for Health Statistics (NCHS) guidelines for pooling eight years of data (four 2-year cycles: 1999–2000, 2001–2002, 2003–2004, and 2009–2010), we constructed the combined examination weights by multiplying the 4-year weights (WTMEC4YR) for 1999–2002 by 1/2, and the 2-year weights (WTMEC2YR) for 2003–2004 and 2009–2010 by 1/4. Additionally, we confirmed that the masked variance units, specifically the strata (SDMVSTRA) and primary sampling units (SDMVPSU), were appropriately harmonised across all included cycles to provide reliable national estimates and accurate standard errors [26].

A stepwise adjusted-weighed multivariable logistic regression model was used to explore the association between multiple reproductive indicators and chronic low back pain. The original model did not adjust for any covariates. Model 1 adjusted for age and race/ethnicity, which are fundamental demographic factors strongly associated with both reproductive patterns and chronic low back pain. Model 2 additionally adjusted for commonly used sociodemographic and lifestyle covariates, including marital status, education level, smoking status, alcohol consumption, poverty income ratio, physical activity, and body mass index, which are frequently considered in population-based studies of musculoskeletal outcomes. Model 3 further incorporated clinical comorbidities that altered the estimated association between reproductive factors and chronic low back pain by more than 10%, including diabetes, cardiovascular disease, hypertension, and hyperlipidaemia, to obtain fully adjusted estimates. This hierarchical modelling approach allowed evaluation of the stability of associations across increasing levels of adjustment while minimising overadjustment and enhancing comparability with prior studies.

Additionally, a restricted cubic spline model [27] was employed to examine the dose-response relationship between various reproductive factors and chronic low back pain. Restricted cubic spline regression is a flexible modelling approach that allows the association between a continuous exposure and an outcome to vary across different exposure levels without imposing a strict linear assumption. Trend tests assessed linear associations for continuous variables. Restricted cubic spline (RCS) models, integrated with survey weights, examined dose-response relationships using 4 knots (5th, 35th, 65th, and 95th percentiles). Reference points (OR = 1.0) were set at 3 for gravidity, 21 for age at first birth, and 30 for age at last birth. Wald tests evaluated nonlinearity. For nonlinear associations (*P* for nonlinearity <0.05), a two-piecewise linear regression with a grid search was applied to identify the threshold (inflection point) that maximised the model likelihood.

Sensitivity analyses were performed for all models included. To ensure the robustness of the results, multiple imputation methods [28] were used to handle missing covariate values, generating five imputed data sets, and the same analyses were repeated. Multiple imputation was conducted under the assumption that data were missing at random. Each imputed data set was analysed using identical statistical models, and parameter estimates were combined according to Rubin’s rules to generate pooled odds ratios and corresponding 95% CIs. Consistency between results from the imputed analyses and complete-case analyses was used to evaluate the potential influence of missing data on the study findings.

Age and BMI were treated as confounding variables in all adjusted models. Additionally, subgroup analyses by age and BMI were performed to assess potential effect modification, given that chronic low back pain in women exhibits marked heterogeneity across the life course and body composition. Age- and BMI-related differences in spinal biomechanics, hormonal milieu, and metabolic burden may modify the association between reproductive history and CLBP. All analyses were two-sided, and differences were considered statistically significant at *P* < 0.05. All statistical analyses were performed using *R*, version 4.0.3 (R Foundation for Statistical Computing, Vienna, Austria).

## RESULTS

This study was based on the NHANES 1999–2004 and 2009–2010 survey cycles, and statistical analysis was performed on gravidity, parity, age at first live birth, and age at last live birth in four cross-sectional study designs. The baseline characteristics of the subjects grouped by chronic low back pain are shown in **Table 1** and Tables S3 and S4 in the **Online Supplementary Document**. Compared with the never pregnant group, CLBP patients had a younger age at first live birth (22.01 *vs.* 23.05 years, *P* < 0.001) and a higher proportion of first births occurring at ≤16 years of age; they also had an earlier age at last live birth and a higher proportion of completing childbearing by ≤24 years of age (**Table 1**). The average number of gravidity in women with chronic low back pain was 2.96 (standard error 0.06). The distribution of gravidity categories was broadly similar between women with and without CLBP, with gravidity of 2–3 being the most common category in both groups (Table S3 in the **Online Supplementary Document**). The distribution of parity categories was broadly similar between women with and without CLBP, with parity of 2–3 being the most common category in both groups (Table S4 in the **Online Supplementary Document**). Additionally, patients with chronic low back pain were more likely to be non-Hispanic white, married, have a poverty index (PIR) between 1.31 and 3.50, have a high school education or higher, be non-smokers, drink alcohol frequently, have a BMI>30 kg/m^2^, engage in physical activity at a level of 1-599 MET-min/week, and have hypertension.

**Table 1:** Baseline characteristics stratified by LBP status

Gravidity, Mdn (IQR)	Overall, n = 64,658,255	Without CLBP, n = 37,076,890	With CLBP, n = 27,581,366	*P*-value*
Gravidity, n (unweighted) (%)	2.00 (1.00, 4.00)	2.00 (1.00, 4.00)	3.00 (1.00, 4.00)	<0.001
*0*				<0.001
*1*	809 (17.23)	539 (20.23)	270 (13.21)	
*2–3*	618 (12.58)	335 (11.59)	283 (13.90)	
*≥4*	2,143 (41.53)	1,228 (41.86)	915 (41.09)	
Age, Mdn (IQR)	2,057 (28.66)	1,115 (26.32)	942 (31.80)	
Race, n (unweighted) (%)	46.00 (34.00, 59.00)	45.00 (33.00, 58.00)	47.00 (35.00, 60.00)	0.009
*Non-Hispanic White*				0.007
*Non-Hispanic Black*	2,940 (73.68)	1,630 (72.71)	1,310 (74.98)	
*Mexican American*	1,062 (10.61)	608 (10.67)	454 (10.53)	
*Other Hispanic*	1,180 (6.09)	728 (6.57)	452 (5.45)	
*Other race*	265 (5.50)	133 (5.13)	132 (5.99)	
Marry, n (unweighted) (%)	180 (4.12)	118 (4.91)	62 (3.05)	
*Married*				0.13
*Never married/Other*	3,066 (60.07)	1,807 (61.15)	1,259 (58.61)	
PIR, n (unweighted) (%)	2,561 (39.93)	1,410 (38.85)	1,151 (41.39)	
*≤1.30*				<0.001
*1.31–3.50*	1,747 (24.13)	907 (21.00)	840 (28.33)	
*>3.50*	2,155 (36.24)	1,224 (35.32)	931 (37.47)	
Education, n (unweighted) (%)	1,725 (39.64)	1,086 (43.68)	639 (34.21)	
*Less than high school*				<0.001
*High school or equivalent*	1,676 (18.79)	889 (15.97)	787 (22.58)	
*Above high school*	1,366 (26.09)	752 (24.22)	614 (28.59)	
Smoke, n (unweighted) (%)	2,585 (55.12)	1,576 (59.81)	1,009 (48.83)	
*Never*				<0.001
*Former*	3,310 (56.08)	2,014 (59.07)	1,296 (52.05)	
*Current*	1,185 (21.27)	644 (20.36)	541 (22.50)	
Drink, n (unweighted) (%)	1,132 (22.65)	559 (20.57)	573 (25.45)	
*Never*				0.003
*Former*	1,170 (16.88)	697 (16.97)	473 (16.77)	
*Current*	1,129 (17.53)	599 (15.86)	530 (19.77)	
PA (MET-min/week), n (unweighted) (%)	3,328 (65.59)	1,921 (67.17)	1,407 (63.46)	
*0*				<0.001
*1–599*	1,911 (27.32)	1,045 (25.57)	866 (29.67)	
*≥600*	2,181 (43.79)	1,340 (47.53)	841 (38.76)	
BMI (kg/m2), n (unweighted) (%)	1,535 (28.89)	832 (26.91)	703 (31.57)	
*<18.5*				<0.001
*18.5–24.9*	110 (2.60)	59 (2.34)	51 (2.96)	
*25–29.9*	1,703 (34.80)	1,079 (38.34)	624 (30.03)	
*>30*	1,699 (28.41)	995 (29.01)	704 (27.60)	
DM, n (unweighted) (%)	2,115 (34.19)	1,084 (30.31)	1,031 (39.41)	
*No*				<0.001
*Yes*	4,852 (90.70)	2,834 (92.37)	2,018 (88.47)	
Hypertension, n (unweighted) (%)	775 (9.30)	383 (7.63)	392 (11.53)	
*No*				<0.001
*Yes*	3,112 (62.64)	1,865 (65.32)	1,247 (59.03)	
Hyperlipidemia, n (unweighted) (%)	2,515 (37.36)	1,352 (34.68)	1,163 (40.97)	
*No*				0.023
*Yes*	4,126 (70.47)	2,322 (68.84)	1,804 (72.66)	
CVD, n (unweighted) (%)	1,501 (29.53)	895 (31.16)	606 (27.34)	
*No*				<0.001
*Yes*	5,039 (91.60)	2,952 (93.54)	2,087 (88.98)	

BMI – Body mass index, CI – confidence interval, CLBP – chronic low back pain, CVD – cardiovascular disease, DM – diabetes mellitus, OR – odds ratio, PA – physical activity, PIR – poverty income ratio

*Wilcoxon rank-sum test for complex survey samples; χ^2^ test with Rao & Scott's second-order correction.

**Figure 2:**
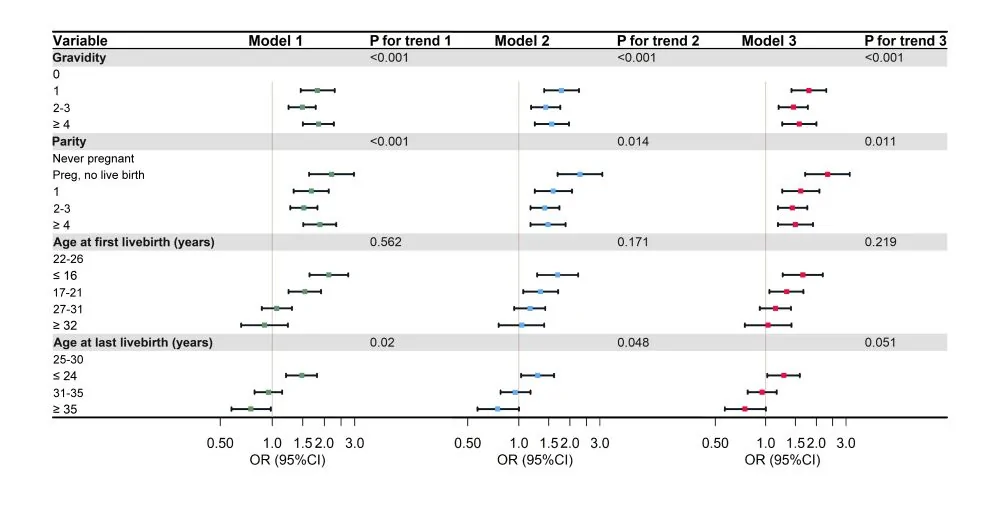
Forest Plot of the logistic regression analyses results for the association between gravidity, parity, age at first live birth, age at last live birth and LBP. Model 1: Adjusted for age, race. Model 2: Adjusted for the variable in Model 1 plus marriage status, education level, smoke, alcohol consumption, poverty income ratio, physical activity, body mass index. Model 3: Adjusted for the variable in Model 2 plus comorbidities such as DM, CVD, hypertension, hyperlipidaemia. CI – confidence interval, CLBP – chronic low back pain, CVD – cardiovascular disease DM – diabetes mellitus, OR – odds ratio, Preg – pregnancy, Ref – reference.

**Figure 3:**
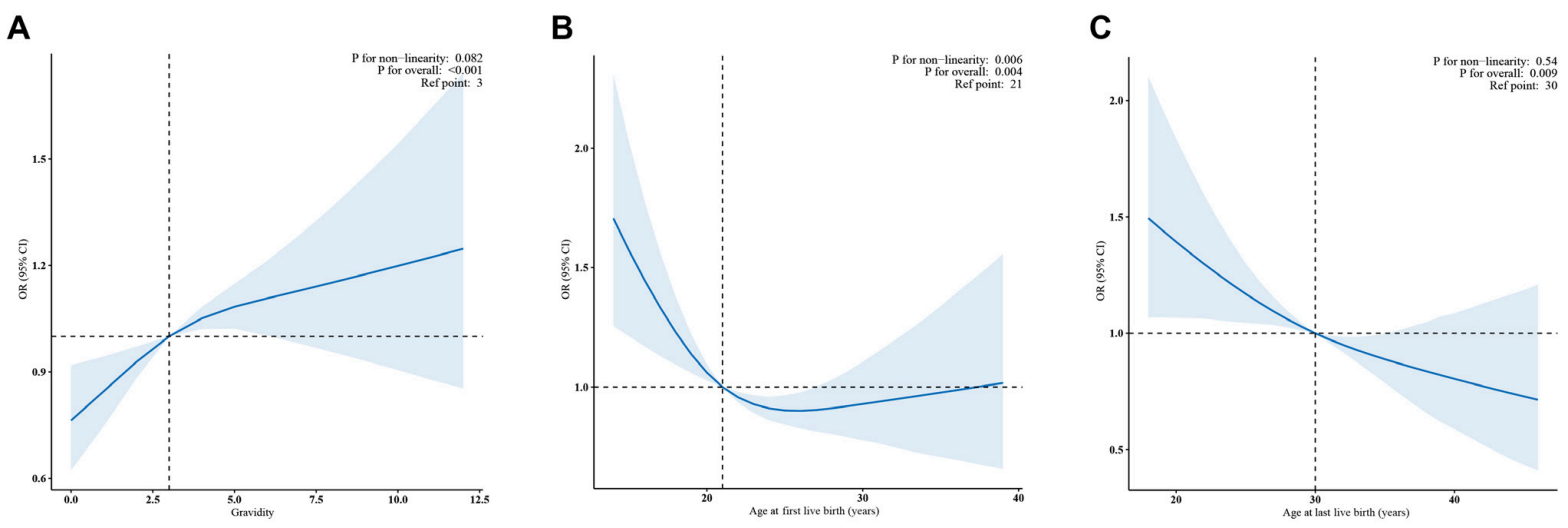
The RCS curve of the association of gravidity (Panel A), age at first live birth (Panel B), age at last live birth (Panel C) and prevalence of Chronic low back pain. RCS – restricted cubic spline, OR – odds ratio, CI – confidence interval, Ref – reference.

This study further examined the associations between gravidity, parity, age at first live birth, age at last live birth, and the odds of CLBP using a multivariable logistic regression model (**Table 2** and **Figure 2**). In the unadjusted model, gravidity (OR = 1.08; 95% CI = 1.05–1.10, *P* < 0.001) and parity (OR = 1.07; 95% CI = 1.04–1.11, *P* < 0.001) were significantly associated with higher odds of CLBP, while age at first live birth (OR = 0.96; 95% CI = 0.94–0.97, *P* < 0.001) and age at last live birth (OR = 0.97; 95% CI = 0.95–0.98, *P* < 0.001) were negatively associated with the odds of CLBP. The results remained consistent after adjusting for all covariates. Compared with women who had never been pregnant, women who had been pregnant only once had an 80% higher prevalence of chronic low back pain (OR = 1.80; 95% CI = 1.42–2.29, *P* < 0.001). Dose-response relationships were further visualised using restricted cubic splines (**Figure 3**). For gravidity, a primarily linear association was observed: P for nonlinearity = 0.082 (**Figure 3**, Panel A). Notably, among women with a history of pregnancy without live births, the odds of CLBP were significantly elevated (OR = 2.33; 95% CI = 1.71–3.16, *P* < 0.001), suggesting that pregnancy experience itself may be associated with higher odds of low back pain even without childbirth. After analysing the age at first live birth, we observed a significant nonlinear relationship between this variable and the odds of chronic low back pain: P for nonlinearity = 0.006 (**Figure 3**, Panel B). Using a two-piecewise linear regression model, a threshold effect was identified at 21 years. Using women who gave birth for the first time at the reference point of 21 years (OR = 1.0), women who gave birth for the first time at ≤16 years of age had a significantly higher odds of CLBP (OR = 1.66; 95% CI = 1.26–2.18, *P* < 0.001). In contrast, no significant difference was observed between women who had their first live birth at ≥32 years of age and the reference group (OR = 1.03; 95% CI = 0.75–1.42, *P* = 0.86). For the analysis of age at last live birth, which included all women with at least one live birth (where age at first and last birth were identical for primiparous women), the results showed that compared with women who had their last live birth between 25 and 30 years of age, women who completed their last live birth at ≤24 years of age had a 28% higher odds of CLBP (OR = 1.28; 95% CI = 1.02–1.59, *P* < 0.05); no significant differences were observed in other age groups, showing a linear trend: P for nonlinearity = 0.54 (**Figure 3**, Panel C).

**Table 2:** Associations of gravidity, parity, age at first live birth, and age at last live birth with chronic low back pain

Variable	Total	CLBP (%)	Non-adjusted OR (95% CI)	Model 1* OR (95% CI)	Model 2* OR (95% CI)	Model 3* OR (95% CI)
Gravidity	5,627	2,410 (42.8)	1.08 (1.05, 1.10)†	1.08 (1.05,1.11)†	1.05 (1.02, 1.08)‡	1.05 (1.02, 1.08)‡
*0*	809	270 (33.4)	Ref	Ref	Ref	Ref
*1*	618	283 (45.8)	1.84 (1.46, 2.31)†	1.82 (1.46, 2.29)†	1.78 (1.41, 2.26)†	1.80 (1.42, 2.29)†
*2-3*	2,143	915 (42.7)	1.50 (1.26, 1.79)†	1.49 (1.24, 1.78)†	1.44 (1.18, 1.75)†	1.46 (1.19, 1.78)†
*≥4*	2,057	942 (45.8)	1.85 (1.52, 2.26)†	1.85 (1.50, 2.27)†	1.56 (1.24, 1.97)†	1.58 (1.25, 2.00)†
Trend. test	5,627	2,410 (42.8)	1.15 (1.09, 1.21)†	1.17 (1.11, 1.24)†	1.11 (1.05, 1.18)‡	1.11 (1.05, 1.18)‡
						
Parity	5,611	2,396 (42.7)	1.07 (1.04, 1.11)†	1.07 (1.04, 1.11)†	1.03 (0.99, 1.07)	1.03 (0.99, 1.07)
Never Pregnant	806	270 (33.4)	Ref	Ref	Ref	Ref
Pregnant, no live birth	198	92 (46.5)	2.19 (1.61, 2.98)†	2.20 (1.63, 2.96)†	2.29 (1.69, 3.10)†	2.33 (1.71, 3.16)†
*1*	811	364 (44.9)	1.69 (1.34, 2.13)†	1.68 (1.33, 2.12)†	1.59 (1.24, 2.05)†	1.61 (1.25, 2.08)†
*2–3*	2,485	1,077 (43.3)	1.54 (1.29, 1.84)†	1.52 (1.27, 1.82)†	1.42 (1.17, 1.73)†	1.44 (1.18, 1.76)†
*≥4*	1,308	593 (45.3)	1.89 (1.53, 2.34)†	1.88 (1.51, 2.34)†	1.48 (1.17, 1.88)‡	1.50 (1.18, 1.91)‡
Trend. test	5,611	2,396 (42.7)	1.11 (1.06, 1.16)†	1.13 (1.08, 1.18)†	1.08 (1.03, 1.14)§	1.09 (1.03, 1.14)§
						
Age at first live birth (years)	4,582	2,024 (44.2)	0.96 (0.94, 0.97)†	0.96 (0.94, 0.97)†	0.98 (0.96, 1.00)§	0.98 (0.96, 1.00)§
*22–26*	1,275	499 (39.1)	Ref	Ref	Ref	Ref
*≤16*	675	366 (54.2)	2.03 (1.58, 2.61)†	2.12 (1.64, 2.74)†	1.69 (1.28, 2.23)†	1.66 (1.26, 2.18)†
*17–21*	1,831	849 (46.4)	1.51 (1.22, 1.87)†	1.54 (1.24, 1.91)†	1.34 (1.06, 1.70)§	1.33 (1.05, 1.67)§
*27–31*	560	219 (29.1)	1.07 (0.88, 1.30)	1.06 (0.87, 1.30)	1.16 (0.94, 1.43)	1.14 (0.92, 1.41)
*≥32*	238	90 (27.8)	0.90 (0.67, 1.23)	0.90 (0.66, 1.23)	1.04 (0.76, 1.41)	1.03 (0.75, 1.42)
Trend. test	4,579	2,023 (44.2)	0.83 (0.78, 0.87)§	0.81 (0.76, 0.86)§	0.87 (0.82, 0.93)§	0.88 (0.82, 0.94)§
						
Age at last live birth (years)	4,582	2,024 (44.2)	0.97 (0.95, 0.98)†	0.97 (0.95, 0.98)†	0.97 (0.95, 0.99)‡	0.97 (0.95, 0.99)‡
*25–30*	1,122	487 (43.4)	Ref	Ref	Ref	Ref
*≤24*	764	401 (52.5)	1.45 (1.19, 1.78)†	1.48 (1.20, 1.81)†	1.29 (1.03, 1.61)§	1.28 (1.02, 1.59)§
*31–35*	1,035	443 (42.8)	0.96 (0.80, 1.15)	0.95 (0.79, 1.14)	0.95 (0.78, 1.17)	0.95 (0.78, 1.16)
*≥35*	858	332 (38.7)	0.78 (0.60, 1.01)	0.75 (0.58, 0.98)§	0.75 (0.57, 1.00)§	0.75 (0.57, 1.00)
Trend. test	3,779	1,663 (44.0)	0.85 (0.80, 0.90)§	0.85 (0.80, 0.91)§	0.87 (0.81, 0.93)§	0.87 (0.82, 0.93)§

CLBP – chronic low back pain, CI – confidence interval, CVD – cardiovascular disease, DM – diabetes mellitus, OR – odds ratio, Preg – pregnancy, Ref – reference

*Model 1: Adjusted for age, race. Model 2: Adjusted for the variable in Model 1 plus marriage status, education level, smoke, alcohol consumption, poverty income ratio, physical activity, body mass index. Model 3: Adjusted for the variable in Model 2 plus comorbidities such as DM, CVD, hypertension, hyperlipidaemia.

†*P* < 0.001.

‡*P* < 0.01.

§*P* < 0.05.

This study conducted a sensitivity analysis to assess the robustness of the study results. Multiple imputation methods were used to handle missing covariate values, generating five imputed data sets, and the multivariate analysis was repeated. The results after imputation (**Table 3**) were consistent with the main analysis results, indicating that the study conclusions have good robustness. In addition, subgroup analyses were conducted to assess the consistency of the associations between gravidity (Figure S1, Panel A in the **Online Supplementary Document**), parity (Figure S1, Panel B in the **Online Supplementary Document**), age at first live birth (Figure S2, Panel C in the **Online Supplementary Document**), and age at last live birth (Figure S2, Panel D in the **Online Supplementary Document**) and the odds of chronic low back pain in different population subgroups (Figure S1 and Figure S2 in the **Online Supplementary Document**), and participants were stratified according to age (<40 years/40–59 years/≥60 years) and BMI (<18.5/18.5–24.9/25–29.9/>30 kg/m^2^). The *P*-values for interaction tests in all subgroups were greater than 0.05, suggesting that the associations above are consistent across different subgroups. However, these null findings for interaction should be interpreted with caution, as the statistical power for interaction testing in stratified survey data may be limited, and the use of broad categories for age and BMI might obscure potential heterogeneity within these groups.

**Table 3:** Sensitivity analysis of the associations between gravidity, parity, age at first live birth, age at last live birth and chronic low back pain

Variable	Total	CLBP (%)	Non-adjusted OR (95% CI)	Model 1* OR (95% CI)	Model 2* OR (95% CI)	Model 3* OR (95% CI)
Gravidity	5,627	2,410 (42.8)	1.04 (1.02, 1.06)†	1.06 (1.04, 1.08)†	1.03 (1.01, 1.06)‡	1.03 (1.01, 1.05)‡
*0*	809	270 (33.4)	Ref	Ref	Ref	Ref
*1*	618	283 (45.8)	1.84 (1.46, 2.31)†	1.69 (1.40, 2.05)†	1.72 (1.42, 2.10)†	1.72 (1.42, 2.10)†
*2–3*	2,143	915 (42.7)	1.50 (1.26, 1.79)†	1.55 (1.33, 1.82)†	1.51 (1.28, 1.78)†	1.52 (1.29, 1.80)†
*≥4*	2,057	942 (45.8)	1.85 (1.52, 2.26)†	1.81 (1.54, 2.13)†	1.59 (1.34, 1.89)†	1.58 (1.33, 1.89)†
						
Parity	5,611	2,396 (42.7)	1.03 (1.01, 1.05)‡	1.06 (1.03, 1.08)†	1.02 (1.00, 1.05) ‡	1.02 (0.99, 1.05)
Never Pregnant	806	270 (33.4)	Ref	Ref	Ref	Ref
Pregnant, no live birth	198	92 (46.5)	1.59 (1.21, 2.09)‡	1.76 (1.28, 2.42)†	1.67 (1.26, 2.21)†	1.68 (1.26, 2.22)†
*1*	811	364 (44.9)	1.69 (1.41, 2.02)†	1.64 (1.34, 2.01)†	1.66 (1.38, 1.99)†	1.66 (1.38, 2.00)†
*2–3*	2,485	1077 (43.3)	1.51 (1.29, 1.76)†	1.55 (1.31, 1.84)†	1.48 (1.25, 1.75)†	1.49 (1.26, 1.76)†
*≥4*	1,308	593 (45.3)	1.61 (1.36, 1.90)†	1.78 (1.46, 2.17)†	1.51 (1.35, 1.83)†	1.50 (1.24, 1.82)†
						
Age at first live birth (years)	4,582	2,024 (44.2)	0.97 (0.96, 0.98)†	0.96 (0.94, 0.97)†	0.98 (0.97, 0.99)†	0.98 (0.97, 0.99)‡
*22–26*	1,275	499 (39.1)	Ref	Ref	Ref	Ref
*≤16*	675	366 (54.2)	1.64 (1.39, 1.93)†	1.96 (1.62, 2.38)†	1.45 (1.22, 1.74)†	1.41 (1.18, 1.69)†
*17–21*	1,831	849 (46.4)	1.30 (1.14, 1.48)†	1.38 (1.19, 1.60)†	1.23 (1.07, 1.40)‡	1.20 (1.05, 1.38)‡
*27–31*	560	219 (29.1)	0.99 (0.83, 1.19)	0.97 (0.79, 1.19)	1.02 (0.85, 1.23)	1.01 (0.84, 1.21)
*≥32*	238	90 (27.8)	0.92 (0.72, 1.18)	0.93 (0.70, 1.24)	0.97 (0.75, 1.25)	0.97 (0.76, 1.25)
						
Age at last live birth (years)	4,582	2,024 (44.2)	0.97 (0.97, 0.98)†	0.97 (0.96, 0.98)†	0.97 (0.96, 0.98)†	0.98 (0.97, 0.99)†
*25–30*	1,122	487 (43.4)	Ref	Ref	Ref	Ref
*≤24*	764	401 (52.5)	1.41 (1.19, 1.67)†	1.46 (1.21, 1.76)†	1.34 (1.15, 1.56)†	1.30 (1.09, 1.56)‡
*31–35*	1,035	443 (42.8)	0.97 (0.83, 1.14)	0.97 (0.82, 1.16)	0.97 (0.82, 1.14)	0.98 (0.83, 1.16)§
*≥35*	858	332 (38.7)	0.85 (0.71, 1.01)	0.84 (0.69, 0.91)§	0.86 (0.59, 1.02)	0.84 (0.70, 1.02)
Gravidity	5,627	2,410 (42.8)	1.04 (1.02, 1.06)†	1.06 (1.04, 1.08)†	1.03 (1.01, 1.06)‡	1.03 (1.01, 1.05)‡
*0*	809	270 (33.4)	Ref	Ref	Ref	Ref
*1*	618	283 (45.8)	1.84 (1.46, 2.31)†	1.69 (1.40, 2.05)†	1.72 (1.42, 2.10)†	1.72 (1.42, 2.10)†
*2–3*	2,143	915 (42.7)	1.50 (1.26, 1.79)†	1.55 (1.33, 1.82)†	1.51 (1.28, 1.78)†	1.52 (1.29, 1.80)†

CLBP – chronic low back pain, CI – confidence interval, CVD – cardiovascular disease, DM – diabetes, OR – odds ratio, Preg – pregnancy, Ref – reference

*Model 1: Adjusted for age, race. Model 2: Adjusted for the variable in Model 1 plus marriage status, education level, smoke, alcohol consumption, poverty income ratio, physical activity, body mass index. Model 3: Adjusted for the variable in Model 2 plus comorbidities such as DM, CVD, hypertension, hyperlipidaemia.

†*P* < 0.001.

‡*P* < 0.01.

§*P* < 0.05.

## DISCUSSION

To our knowledge, this study is among the first to systematically investigate the associations between female reproductive factors and the prevalence of chronic low back pain (CLBP) using nationally representative NHANES data. We identified several high-risk profiles, including primiparity, high gravidity/parity (≥4), early childbearing, and pregnancy without live birth. Notably, while the associations for continuous gravidity and parity were statistically significant, the modest ORs (1.08 and 1.07) suggest a limited individual impact per unit increase. These findings likely reflect a cumulative burden in high-parity women, though residual confounding from unmeasured factors, such as specific occupational physical demands, cannot be excluded.

The observed link between high gravidity and CLBP may relate to cumulative physiological stress. Pregnancy involves significant hormonal fluctuations, such as increased relaxin levels [29,30] acting on oestrogen receptors [31], potentially leading to ligaments laxity [6,32,33] reduced lumbar stability [8,34,35]. Additionally, shifts in the centre of gravity and postural compensation correlate with increased spinal load [36,37], potentially contributing to the development of chronic pain. While high parity may result in repeated accumulation of these structural changes, the lower odds at moderate fertility (2–3 births) suggest a potential ‘moderate pregnancy window’ where adaptive behaviours might mitigate lumbar strain.

The childbirth process (particularly live birth) may impose further biomechanical stress [13]. Vaginal delivery involves mechanical stress on anatomical structures, while caesarean sections potentially interfere with core muscle groups, influencing the lumbar support system. Postpartum caregiving activities may further exacerbate this stress [13]. Notably, pregnancy without live birth (encompassing miscarriage, induced abortion, ectopic pregnancy, and stillbirth) showed the highest odds of CLBP. While this heterogeneous category is under-researched [38,39], our results suggest it may serve as a significant clinical correlate. While not directly measured, several hypothetical pathways warrant investigation:

• sudden hormonal shifts might influence disc metabolism [40];

• certain clinical procedures could theoretically alter the local physiological environment [41]; and

• psychosocial stress may potentially influence pain perception through neuroendocrine pathways [42,43].

These proposed mechanisms are intended to provide a theoretical framework for future research rather than confirmed pathways.

Regarding reproductive timing, younger age at first and last live birth were associated with higher CLBP prevalence, consistent with Mendelian randomisation and cross-sectional evidence [13,34,44]. Young women may be more sensitive to hormonal changes [8,34,45], and face higher odds due to an immature spine experiencing biomechanical stress [13,39,46]. Higher prevalence in young mothers has been reported in both cross-sectional [47,48], and prospective studies [46,49]. Managing birth intervals and focusing on reproductive health in young women is essential to reduce cumulative lumbar burden.

Furthermore, the potential for reverse causation must be explicitly considered when interpreting these findings. It is plausible that pre-existing chronic pain or underlying health conditions may influence a woman's reproductive choices, including the decision to pursue further pregnancies or the timing of childbirth. Therefore, the observed associations may partly reflect the impact of pain on reproductive trajectories rather than solely the reverse. Our results should be viewed as identifying complex, potentially bidirectional links between reproductive history and lumbar health that require future longitudinal data to untangle.

### Limitations

Several limitations should be acknowledged. First, reproductive history and CLBP data were self-reported, introducing potential recall or diagnostic bias. While the 12-month recall window for CLBP captures current symptoms, the cross-sectional design precludes establishing a definitive temporal sequence and introduces the possibility of reverse causation, as discussed above. Second, the ‘pregnancy without live birth’ category is clinically heterogeneous, encompassing miscarriage, induced abortion, ectopic pregnancy, and stillbirth, which could not be differentiated due to data limitations. Third, while the NHANES sample is robust, the exclusion of participants with missing data and specific survey cycles may influence the generalisability; thus, the findings should be interpreted with appropriate caution regarding the broader non-institutionalised population. Nonetheless, this study provides a robust epidemiological foundation for identifying vulnerable populations and suggests the potential value of considering reproductive history during clinical assessments. While prospective validation is required to further refine these associations, this study offers a foundational step toward understanding the integration of reproductive history into women’s lumbar health management. Future studies should validate causal pathways using prospective cohorts, explore hypothetical biological mechanisms, including inflammatory markers, hormone profiling, and neuroendocrine factors, and develop and evaluate multidimensional interventions tailored to these mechanisms.

## CONCLUSIONS

Utilising nationally representative NHANES data, this study systematically evaluated the associations between key reproductive history indicators and CLBP among a sample of the US women. We identified several potential high-risk profiles, including a first live birth, high gravidity/parity (≥4), early childbearing, high parity with an early last live birth, and a history of pregnancy without live birth. Notably, to our knowledge, this study is among the first to observe a significant association between pregnancy without live birth and higher CLBP prevalence in a large, population-based sample, providing preliminary insights for women’s lumbar health management. While based on a representative design, the generalisability of these findings should be interpreted with caution due to the reliance on self-reported data and the exclusion of participants with incomplete records. Overall, the results provide preliminary evidence to support future research into the early identification of women at higher CLBP burden and underscore the importance of further exploring the integration of reproductive history into clinical assessment of chronic low back pain through prospective studies.

## Additional material


Online Supplementary Document

## Data Availability

**Data availability:** The data set supporting the conclusions of this article is available in the NHANES repository, https://www.cdc.gov/nchs/nhanes.
