# Supplementary material for: Association between reproductive history and chronic low back pain in the US women: a cross-sectional study
Source: J Glob Health. 2026 Aug 28;16:04238. doi: 10.7189/jogh.16.04238 (PMC13522905; doi:10.7189/jogh.16.04238)
Supplement: Supplementary file 1 [file jogh-16-04238-s001.pdf]

Supplement to: Zhang Y, Zhao L, Hu D, Shen X, Dong X, Lu Y. Association between reproductive history and chronic low back pain in the US women: a cross-sectional study. J Glob Health. 2026;16:04238.

Supplementary Figure 1 Forest plot of subgroup analysis of gravidity(A) and parity(B).

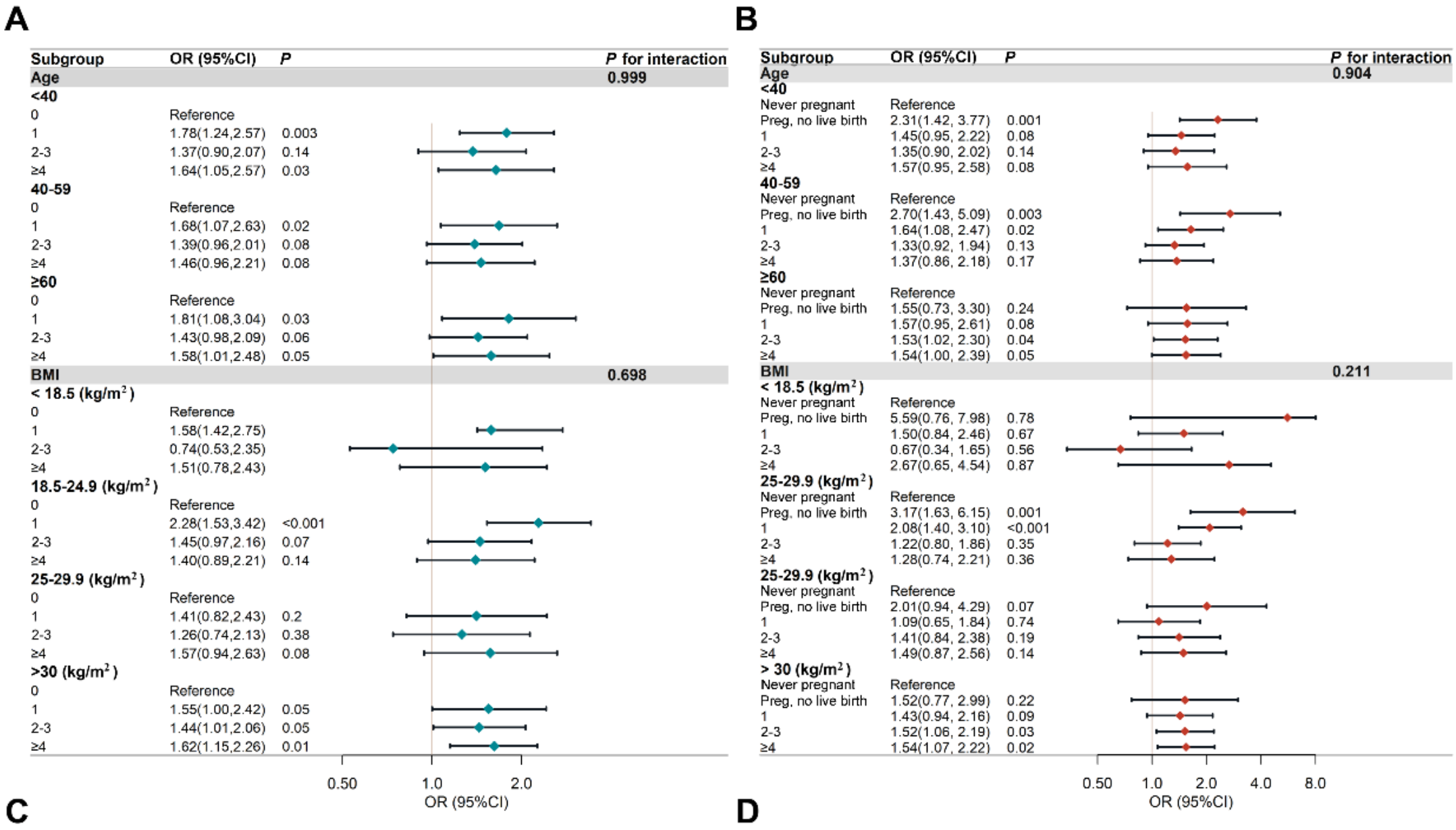

Abbreviations: BMI – body mass index, CI – confidence interval, CLBP – chronic low back pain, CVD – cardiovascular disease, DM – diabetes, OR – odds ratio, PA – physical activity, PIR – poverty income ratio

Supplementary Figure 2 Forest plot of subgroup analysis of age at first live birth(C) and age at last live birth(D).

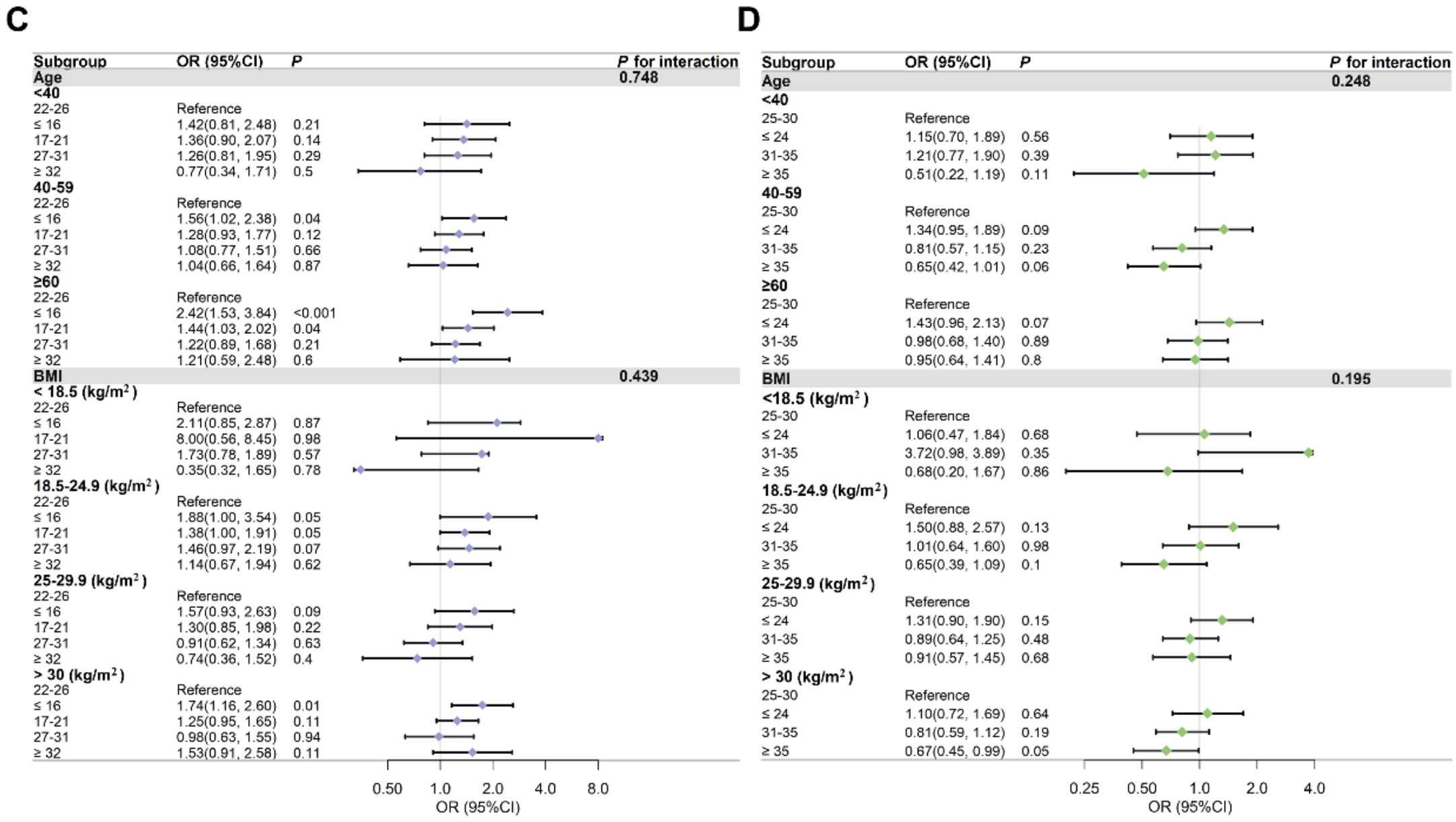

Abbreviations: BMI – body mass index, CI – confidence interval, CLBP – chronic low back pain, CVD – cardiovascular disease, DM – diabetes, OR – odds

ratio, PA – physical activity, PIR – poverty income ratio

Supplementary Table 1 Adherence to JoGH's GRABDROP guidelines items

**JoGH guideline items**
1. Please list all papers published by each co-author in previous 3 years that were based on secondary analysis of a big data repository
[1] Shen X, Hu D, Lu Y, Zhao L, Yuan H, Sun X. Association between weight-adjusted waist and incidence of low back pain: evidence from the China Health and Retirement Longitudinal Study. Aging Clin Exp Res. 2026 Apr 14. doi: 10.1007/s40520-026-03351-1. Epub ahead of print. PMID: 41979811.<br>[2] Hu D, Zhang Y, Liu X, Yang X, Liang X, Hu X, Yuan H, Zhao C. Sleeping <6.55 h per day was associated with a higher risk of low back pain in adults aged over 50 years: a Korean nationwide cross-sectional study. Front Public Health. 2024 Sep 20;12:1429495. doi: 10.3389/fpubh.2024.1429495. PMID: 39371204; PMCID: PMC11449761.
2. Please explain the key elements of your study design and the use of the available datasets that make your study an original scientific contribution
This study employs a cross-sectional design using the NHANES database to systematically evaluate the association between a comprehensive set of reproductive factors and Chronic Low Back Pain (CLBP). While previous research has touched upon parity, our study is original in its integration of miscarriage history, age at first live birth, and age at last live birth into a single analytical framework. It provides pioneering evidence on how specific reproductive "high-risk profiles" (e.g., non-live birth outcomes and early childbearing) correlate with long-term lumbar health in a nationally representative U.S. sample.
3. Please list all publications that addressed similar research questions in the same dataset and indicate where you cited them in your paper
We performed a systematic search on PubMed using keywords including "NHANES", "low back pain", and "reproductive history". Our search did not identify any prior publications within the NHANES database that specifically investigated the associations between these reproductive factors and CLBP. Consequently, no similar studies using this specific dataset are cited in this manuscript. Our findings thus represent an original contribution to the existing literature.
4. Please explain how you addressed multiple testing through an appropriately rigorous statistical threshold and indicate this in the methods section
Multiple testing correction was not applied because this study was hypothesis-driven and focused on a single primary outcome (CLBP). The reproductive factors analyzed were pre-specified based on clinical relevance rather than exploratory data mining. Therefore, a standard significance threshold of $P<0.05$ was considered appropriate for this focused analysis.
5. Please declare to what extent have AI chatbots been used in developing your paper and to which parts of the paper did they contribute

AI tools were used solely for language polishing, grammatical corrections, and improving the overall clarity and readability of the manuscript. No AI was used for data collection, statistical analysis, or the generation of the core scientific content.

Supplementary Table 2 Definitions and classification of covariates.

Covariates	Definition	Classification
**Age**		1- < 40<br>2- 40-59<br>3- ≥ 60
**Race**		1- Non-Hispanic White,<br>2- Non-Hispanic Black<br>3- Mexican American<br>4- Other Hispanic<br>5- Other Race-including Multi-Racial
**Marry**	2- Never married/Other: widowed, divorced, or separated individuals	1- Married/ Living with partner<br>2- Never married/Other: widowed, divorced, or separated individuals
**Education**	1- Less than high school: Less Than 9th Grade and 9-11th Grade (Includes 12th grade with no diploma)<br>2- High school or equivalent: High school graduate/GED or equivalent<br>3- Above high school:some college or AA degree and college graduate or above	1- Less than high school<br>2- High school or equivalent<br>3- Above high school
**PIR** [1]	poverty-to-income ratio (PIR) is an index of income related to household needs, by calculating annual changes in household size and cost of living and tracking the consumer price index from household income and federally determined poverty thresholds	1- ≤1.30<br>2- 1.31-3.50<br>3- >3.50
**BMI** [2]	Height was measured using a stadiometer, and weight was measured using a digital scale following standard procedures at the MEC. BMI was calculated using weight in kilograms divided by height in meters squared.	1- < 18.5<br>2- 18.5-24.9<br>3- 25-29.9<br>4- > 30
**PA** [3]	MET values vary with the type of	1- 0

	exercise, and the NHANES offers the recommended MET values for each PA. PA was based on the MET values of type, frequency, and duration of activities per week, which was calculated using the following formula: PA (MET-min/week) = MET × weekly frequency × duration of each PA.	2- 1-599<br>3- ≥ 600
**Drink** [4]	1. never: had <12 drinks in lifetime<br>2. former: had ≥12 drinks in 1 year and did not drink last year, or did not drink last year but drank ≥12 drinks in lifetime<br>3. current alcohol use: ≤1 drinks per day for females, ≤2 drinks per day for males	1- never<br>2- former<br>3- current
**Smoke** [5]	1. never: smoked less than 100 cigarettes in life<br>2. former: smoked more than 100 cigarettes in life and smoke not at all now<br>3. now: smoked moth than 100 cigarettes in life and smoke some days or every day	1- never<br>2- former<br>3- current
**Hypertension** [6]	Meeting any single criterion establishes diagnosis：<br>1. Self-reported physician-diagnosed hypertension<br>2. Current use of antihypertensive medication(s)<br>3. Mean systolic blood pressure (SBP) ≥140 mmHg or mean diastolic blood pressure (DBP) ≥90 mmHg	0- no<br>1- yes
**Hyperlipidemia** [7]	Diagnosis established by meeting ≥1 criterion)<br>1. Current use of antihyperlipidemic agents<br>2. Hypertriglyceridemia: Serum triglycerides ≥150 mg/dL (1.7 mmol/L)	0- no<br>1- yes

	3. Hypercholesterolemia defined as:Total cholesterol ≥200 mg/dL (5.2 mmol/L) or LDL-cholesterol ≥130 mg/dL (3.4 mmol/L) or HDL-cholesterol <40 mg/dL (1.0 mmol/L)	
**CVD** [8]	The diagnosis was established when a history of any of the following physician-diagnosed cardiovascular conditions was self-reported: coronary heart disease, angina, stroke, myocardial infarction, or congestive heart failure.	0- no<br>1- yes
**DM** [9]	The diagnostic criteria for diabetes are:<br>1. doctor told you have diabetes,<br>2. glycohemoglobin HbA1c(%) >= 6.5,<br>3. fasting glucose (mmol/l) >= 7.0,<br>4. random blood glucose (mmol/l) >= 11.1,<br>5. two-hour OGTT blood glucose (mmol/l) >= 11.1,<br>6. Use of diabetes medication or insulin	0- no<br>1- yes

Supplementary Table 3 Baseline characteristics of the study population, stratified by chronic low back pain status.

Characteristic	Overall (N = 64,658,255)	Without CLBP (N = 37,076,890)	With CLBP (N = 27,581,366)	***P***[1]
Gravidity				<0.001
Mean (SE)	2.72 (0.04)	2.55 (0.05)	2.96 (0.06)	
Median (IQR)	2.00 (1.00, 4.00)	2.00 (1.00, 4.00)	3.00 (1.00, 4.00)	
Gravidity, n (unweighted) (%)				<0.001
0	809 (17.23%)	539 (20.23%)	270 (13.21%)	
1	618 (12.58%)	335 (11.59%)	283 (13.90%)	
2-3	2,143 (41.53%)	1,228 (41.86%)	915 (41.09%)	
≥ 4	2,057 (28.66%)	1,115 (26.32%)	942 (31.80%)	
Age				0.009
Median (IQR)	46.00 (34.00, 59.00)	45.00 (33.00, 58.00)	47.00 (35.00, 60.00)	
Race, n (unweighted) (%)				0.007
Non-Hispanic White	2,940 (73.68%)	1,630 (72.71%)	1,310 (74.98%)	
Non-Hispanic Black	1,062 (10.61%)	608 (10.67%)	454 (10.53%)	
Mexican American	1,180 (6.09%)	728 (6.57%)	452 (5.45%)	
Other Hispanic	265 (5.50%)	133 (5.13%)	132 (5.99%)	
Other Race	180 (4.12%)	118 (4.91%)	62 (3.05%)	
Marry, n (unweighted) (%)				0.13

Characteristic	Overall (N = 64,658,255)	Without CLBP (N = 37,076,890)	With CLBP (N = 27,581,366)	***P***[1]
Married	3,066 (60.07%)	1,807 (61.15%)	1,259 (58.61%)	
Never married/Other	2,561 (39.93%)	1,410 (38.85%)	1,151 (41.39%)	
PIR, n (unweighted) (%)				<0.001
≤ 1.30	1,747 (24.13%)	907 (21.00%)	840 (28.33%)	
1.31-3.50	2,155 (36.24%)	1,224 (35.32%)	931 (37.47%)	
> 3.50	1,725 (39.64%)	1,086 (43.68%)	639 (34.21%)	
Education, n (unweighted) (%)				<0.001
Less than high school	1,676 (18.79%)	889 (15.97%)	787 (22.58%)	
High school or equivalent	1,366 (26.09%)	752 (24.22%)	614 (28.59%)	
Above high school	2,585 (55.12%)	1,576 (59.81%)	1,009 (48.83%)	
Smoke, n (unweighted) (%)				<0.001
Never	3,310 (56.08%)	2,014 (59.07%)	1,296 (52.05%)	
Former	1,185 (21.27%)	644 (20.36%)	541 (22.50%)	
Current	1,132 (22.65%)	559 (20.57%)	573 (25.45%)	
Drink, n (unweighted) (%)				0.003
Never	1,170 (16.88%)	697 (16.97%)	473 (16.77%)	
Former	1,129 (17.53%)	599 (15.86%)	530 (19.77%)	
Current	3,328 (65.59%)	1,921 (67.17%)	1,407 (63.46%)	

Characteristic	Overall (N = 64,658,255)	Without CLBP (N = 37,076,890)	With CLBP (N = 27,581,366)	***P***[1]
PA(MET-min/week), n (unweighted) (%)				<0.001
0	1,911 (27.32%)	1,045 (25.57%)	866 (29.67%)	
1-599	2,181 (43.79%)	1,340 (47.53%)	841 (38.76%)	
≥ 600	1,535 (28.89%)	832 (26.91%)	703 (31.57%)	
BMI (kg/m^2), n (unweighted) (%)				<0.001
< 18.5	110 (2.60%)	59 (2.34%)	51 (2.96%)	
18.5-24.9	1,703 (34.80%)	1,079 (38.34%)	624 (30.03%)	
25-29.9	1,699 (28.41%)	995 (29.01%)	704 (27.60%)	
> 30	2,115 (34.19%)	1,084 (30.31%)	1,031 (39.41%)	
DM, n (unweighted) (%)				<0.001
No	4,852 (90.70%)	2,834 (92.37%)	2,018 (88.47%)	
Yes	775 (9.30%)	383 (7.63%)	392 (11.53%)	
Hypertension, n (unweighted) (%)				<0.001
No	3,112 (62.64%)	1,865 (65.32%)	1,247 (59.03%)	
Yes	2,515 (37.36%)	1,352 (34.68%)	1,163 (40.97%)	
Hyperlipidemia, n (unweighted) (%)				0.023
No	1,501 (29.53%)	895 (31.16%)	606 (27.34%)	
Yes	4,126 (70.47%)	2,322 (68.84%)	1,804 (72.66%)	
CVD, n (unweighted) (%)				<0.001
No	5,039 (91.60%)	2,952 (93.54%)	2,087 (88.98%)	

Characteristic	**Overall**<br>(N = 64,658,255)	**Without CLBP**<br>(N = 37,076,890)	**With CLBP**<br>(N = 27,581,366)	***P***[1]
Yes	588 (8.40%)	265 (6.46%)	323 (11.02%)	

[1]Wilcoxon rank-sum test for complex survey samples; chi-squared test with Rao & Scott's second-order correction.

a: A gravidity of zero, b: A parity of zero

Abbreviations: NLB Number of Live Births, PIR Poverty Income Ratio, PA Physical activity, BMI body mass index, OR Odds ratio, CI Confidence interval, DM diabetes, CVD Cardiovascular disease, CLBP chronic low back pain

Supplementary Table 4

**Characteristic**	**Overall,** (N = 64,477,257)	**Without CLBP** (N = 37,042,840)	**With CLBP** (N = 27,434,417)	***P***[1]
Parity, Median (IQR)	3.00 (2.00, 4.00)	3.00 (2.00, 4.00)	3.00 (2.00, 4.00)	<0.001
Parity, n (unweighted) (%)				<0.001
0[a]	809 (17.28%)	539 (20.25%)	270 (13.28%)	
0[b]	198 (4.10%)	106 (3.46%)	92 (4.97%)	
1	811 (15.81%)	447 (15.12%)	364 (16.75%)	
2-3	2,485 (47.20%)	1,408 (47.02%)	1,077 (47.44%)	
≥ 4	1,308 (15.61%)	715 (14.15%)	593 (17.56%)	
Age				0.008
Mean (mean ± SD)	47.25 (0.35)	46.67 (0.44)	48.04 (0.43)	
Median (IQR)	46.00 (34.00, 59.00)	45.00 (33.00, 58.00)	47.00 (35.00, 60.00)	
Race, n (unweighted) (%)				0.008
Non-Hispanic White	2,930 (73.65%)	1,627 (72.67%)	1,303 (74.98%)	
Non-Hispanic Black	1,062 (10.64%)	609 (10.69%)	453 (10.56%)	
Mexican American	1,178 (6.10%)	728 (6.58%)	450 (5.45%)	
Other Hispanic	261 (5.48%)	133 (5.14%)	128 (5.94%)	
Other Race	180 (4.13%)	118 (4.92%)	62 (3.07%)	
Marry, n (unweighted) (%)				0.12
married	3,057 (60.01%)	1,805 (61.11%)	1,252 (58.53%)	
Never married/Other	2,554 (39.99%)	1,410 (38.89%)	1,144 (41.47%)	
PIR, n (unweighted) (%)				<0.001

**Characteristic**	**Overall,** (N = 64,477,257)	**Without CLBP** (N = 37,042,840)	**With CLBP** (N = 27,434,417)	***P***[1]
≤ 1.30	1,743 (24.13%)	908 (21.03%)	835 (28.31%)	
1.31-3.50	2,153 (36.32%)	1,224 (35.35%)	929 (37.63%)	
> 3.50	1,715 (39.56%)	1,083 (43.62%)	632 (34.06%)	
Education, n (unweighted) (%)				<0.001
Less than high school	1,676 (18.84%)	889 (15.98%)	787 (22.70%)	
High school or equivalent	1,362 (26.08%)	751 (24.20%)	611 (28.62%)	
Above high school	2,573 (55.08%)	1,575 (59.82%)	998 (48.68%)	
Smoke, n (unweighted) (%)				<0.001
never	3,305 (56.15%)	2,014 (59.13%)	1,291 (52.11%)	
former	1,183 (21.29%)	645 (20.39%)	538 (22.50%)	
current	1,123 (22.57%)	556 (20.48%)	567 (25.39%)	
Drink, n (unweighted) (%)				0.003
never	1,170 (16.93%)	698 (17.00%)	472 (16.83%)	
former	1,128 (17.54%)	599 (15.87%)	529 (19.79%)	
current	3,313 (65.53%)	1,918 (67.13%)	1,395 (63.38%)	
BMI (kg/m^2), n (unweighted) (%)				<0.001
< 18.5	110 (2.61%)	59 (2.34%)	51 (2.98%)	
18.5-24.9	1,699 (34.81%)	1,079 (38.33%)	620 (30.06%)	
25-29.9	1,697 (28.43%)	996 (29.05%)	701 (27.59%)	
> 30	2,105 (34.15%)	1,081 (30.28%)	1,024 (39.37%)	

Characteristic	**Overall**, (N = 64,477,257)	**Without CLBP** (N = 37,042,840)	**With CLBP** (N = 27,434,417)	***P***[1]
PA(MET-min/week), n (unweighted) (%)				<0.001
0	1,908 (27.35%)	1,044 (25.58%)	864 (29.73%)	
1-599	2,180 (43.87%)	1,341 (47.58%)	839 (38.85%)	
≥ 600	1,523 (28.79%)	830 (26.84%)	693 (31.42%)	
CVD, n (unweighted) (%)				<0.001
no	5,023 (91.60%)	2,949 (93.53%)	2,074 (89.00%)	
yes	588 (8.40%)	266 (6.47%)	322 (11.00%)	
DM, n (unweighted) (%)				<0.001
no	4,838 (90.72%)	2,831 (92.35%)	2,007 (88.53%)	
yes	773 (9.28%)	384 (7.65%)	389 (11.47%)	
Hypertension, n (unweighted) (%)				<0.001
no	3,103 (62.68%)	1,864 (65.30%)	1,239 (59.13%)	
yes	2,508 (37.32%)	1,351 (34.70%)	1,157 (40.87%)	
Hyperlipidemia, n (unweighted) (%)				0.021
no	1,497 (29.53%)	895 (31.19%)	602 (27.30%)	
yes	4,114 (70.47%)	2,320 (68.81%)	1,794 (72.70%)	

[1]Wilcoxon rank-sum test for complex survey samples; chi-squared test with Rao & Scott's second-order correction

a: A gravidity of zero, b: A parity of zero

Abbreviations: NLB Number of Live Births, PIR Poverty Income Ratio, PA Physical activity, BMI body mass index, OR Odds ratio, CI Confidence interval, DM diabetes, CVD Cardiovascular disease, CLBP chronic low back pain.

Supplementary Table 5 Multicollinearity diagnostics and Generalized Variance Inflation Factor (GVIF) results for covariates in the multivariable models.

Variable	Coeff (Model I)	OR (%)	Coeff (Full)	OR (%)	GVIF	DF	$GVIF^{1/(2\times Df)}$	collinearity	select	select.VIF
Crude	0.05	Ref.	0.03	Ref.	1.236	1	1.112	0	Ref.	Ref.
Age	0.05	-1.8	0.03	-19.5	1.876	2	1.17	0	Yes	Yes
Race	0.06	20.4	0.02	-27.5	1.459	4	1.048	0	Yes	Yes
Marry	0.05	2.2	0.03	-5.2	1.152	1	1.073	0	No	Yes
PIR	0.03	-24.9	0.04	13.2	1.433	2	1.094	0	Yes	Yes
Education	0.04	-24.9	0.04	11.7	1.518	2	1.11	0	Yes	Yes
Smoke	0.05	-0.8	0.03	7	1.266	2	1.061	0	No	Yes
Drink	0.05	-1.1	0.03	-0.7	1.296	2	1.067	0	No	Yes
CVD	0.04	-13.8	0.03	2.6	1.137	1	1.066	0	Yes	Yes
Hyperlipidemia	0.04	-4.2	0.03	0	1.12	1	1.058	0	No	Yes
Hypertension	0.04	-15.5	0.03	0	1.467	1	1.211	0	Yes	Yes
DM	0.04	-12.8	0.03	1.1	1.172	1	1.083	0	Yes	Yes
BMI	0.04	-14.5	0.03	6.3	1.193	3	1.03	0	Yes	Yes
PA	0.04	-8.6	0.03	2.8	1.124	2	1.03	0	No	Yes

GVIF Diagnostic: Generalized Variance Inflation Factor (GVIF) was used to assess multicollinearity. For variables with multiple degrees of freedom (Df), $GVIF^{1/(2\times Df)}$ is reported. A value <2.0 (equivalent to a standard VIF <4.0) indicates the absence of significant multicollinearity.

Abbreviations: CVD, cardiovascular disease; DM, diabetes mellitus; BMI, body mass index; PA, physical activity; PIR, poverty income ratio.

VIF Screening Result: "Yes" indicates the variable passed the multicollinearity test and is statistically stable for inclusion.
